# Atomic-Resolution Experimental Structural Biology and Molecular Dynamics Simulations of Hyaluronan and Its Complexes

**DOI:** 10.3390/molecules27217276

**Published:** 2022-10-26

**Authors:** Olgun Guvench

**Affiliations:** Department of Pharmaceutical Sciences and Administration, School of Pharmacy, Westbrook College of Health Professions, University of New England, 716 Stevens Avenue, Portland, ME 04103, USA; oguvench@une.edu

**Keywords:** hyaluronan, hyaluronic acid, hyaluronate, flexibility, conformation, crystallography, NMR, molecular dynamics, interaction

## Abstract

This review summarizes the atomic-resolution structural biology of hyaluronan and its complexes available in the Protein Data Bank, as well as published studies of atomic-resolution explicit-solvent molecular dynamics simulations on these and other hyaluronan and hyaluronan-containing systems. Advances in accurate molecular mechanics force fields, simulation methods and software, and computer hardware have supported a recent flourish in such simulations, such that the simulation publications now outnumber the structural biology publications by an order of magnitude. In addition to supplementing the experimental structural biology with computed dynamic and thermodynamic information, the molecular dynamics studies provide a wealth of atomic-resolution information on hyaluronan-containing systems for which there is no atomic-resolution structural biology either available or possible. Examples of these summarized in this review include hyaluronan pairing with other hyaluronan molecules and glycosaminoglycans, with ions, with proteins and peptides, with lipids, and with drugs and drug-like molecules. Despite limitations imposed by present-day computing resources on system size and simulation timescale, atomic-resolution explicit-solvent molecular dynamics simulations have been able to contribute significant insight into hyaluronan’s flexibility and capacity for intra- and intermolecular non-covalent interactions.

## 1. Physical, Chemical, and Biological Properties of Hyaluronan

The glycosaminoglycan hyaluronan is a linear carbohydrate biopolymer composed of a repeating disaccharide of β-D-glucuronate (GlcA) and *N*-acetyl-β-D-glucosamine (GlcNAc) [1]. Unlike other glycosaminoglycans found in human biology, such as chondroitin/dermatan and heparan/heparin, GlcA monosaccharides in hyaluronan are not subject to enzymatic epimerization to α-L-iduronate (IdoA) [1,2]. Furthermore, hyaluronan is not enzymatically sulfated, unlike the glycosaminoglycans chondroitin/dermatan sulfate, heparan sulfate, heparin, or keratan sulfate [1,2]. While these factors make hyaluronan simpler than these other glycosaminoglycans, hyaluronan is nevertheless a large flexible molecule. The size and flexibility of hyaluronan complicate efforts at understanding its atomic-resolution structural biology, which includes interactions and conformational properties relevant to its intrinsic structure as well as to its pairings with other hyaluronan molecules and glycosaminoglycans, with ions, with proteins, with lipids, and with drugs or drug-like molecules. Expanding this structural biology knowledge has the potential to improve mechanistic understanding of hyaluronan biology, which consists principally of contributions to extra- and peri-cellular structure and associated signaling pathways [3,4,5,6,7], although recent findings also point to intracellular roles [8]. Consequences of this structure and signaling have bearing on aging [9,10,11,12,13,14], inflammation [15,16,17,18,19,20,21,22,23], wound healing [24,25,26,27], and cancer [28,29,30,31,32,33,34].

**Figure 1:**
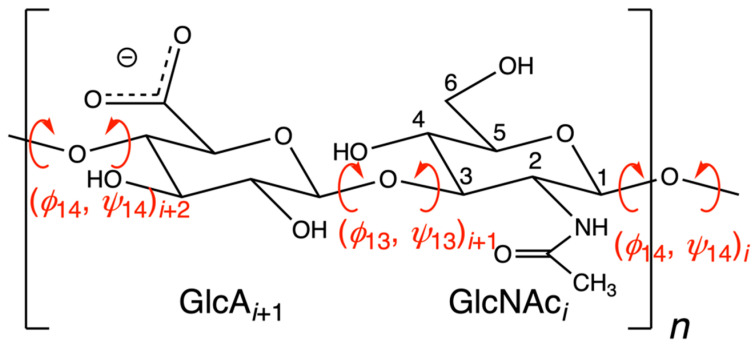
Chemical structure of the glycosaminoglycan biopolymer hyaluronan. GlcA = β-D-glucuronate; GlcNAc = *N*-acetyl-β-D-glucosamine. The GlcA carboxylic acid moiety is expected to be deprotonated at physiological pH and is represented as such. Rotatable dihedrals in β1-4 and β1-3 glycosidic linkages are in red. The index *n* indicates the overall length of the polymer. The index *i* uniquely identifies each monosaccharide in the polymer. IUPAC conventions are used to define (*ϕ*_14_, *ψ*_14_) and (*ϕ*_13_, *ψ*_13_) and assign to each an index *i* [41]. For example, (*ϕ*_14_, *ψ*_14_)*_i_* is defined by (O5*_i_*-C1*_i_*-O4*_i_*_−1_-C4*_i_*_−1_, C1*_i_*-O4*_i_*_−1_-C4*_i_*_−1_-C3*_i_*_−1_) and (*ϕ*_13_, *ψ*_13_)*_i_*_+1_ by (O5*_i_*_+1_-C1*_i_*_+1_-O3*_i_*-C3*_i_*, C1*_i_*_+1_-O3*_i_*-C3*_i_*-C2*_i_*). Numbering of carbon atoms follows the convention for GlcNAc in the figure, with oxygen atoms assuming the number of the carbon to which they are attached. The ring oxygen is considered O5, as it is attached solely to C5 in the linear aldose form of the monosaccharide. Glycosidic linkage oxygen atoms assume the number of the attached carbon atom of the monosaccharide having the lower index *i* in the relevant disaccharide unit.

Hyaluronan polymers found in normal biological fluids and tissues have molecular masses in the range of 1000–8000 kDa [35], which equates to 2600–21,000 repeats of the -4GlcAβ1-3GlcNAcβ1- disaccharide (Figure 1, 2600 < *n* < 21,000). To put this in perspective, this is ~100× more massive than the average eukaryotic protein with mass 55 kDa, assuming an average protein length of 500 amino acids [36] and a weighted average mass of 110 Da per amino acid. The principal source of flexibility for a hyaluronan polymer chain is rotation of dihedrals in the glycosidic linkages (Figure 1, (*ϕ*_14_, *ψ*_14_) and (*ϕ*_13_, *ψ*_13_)). Flexing of the six-membered rings of the constituent monosaccharides provides additional conformational variability in principle; however, GlcA and GlcNAc monosaccharides strongly prefer the ^4^C_1_ ring pucker in an aqueous environment [37,38,39,40], which in turn limits the contribution of ring flexing to overall hyaluronan polymer flexibility.

The large size and the intrinsic flexibility of biological hyaluronan pose obstacles to a comprehensive understanding of its structural and signaling functions across length and timescales. With regard to length scales, on the one hand, hyaluronan non-covalent binding with a given protein partner involves a short length (Figure 1, *n* < 10) of the larger hyaluronan polymer, with upper limits on this length imposed by the size of binding interfaces available on proteins [42]. On the other hand, because of its large size, a single hyaluronan polymer can simultaneously bind to many protein molecules, with biological function dependent upon such polyvalent binding. Examples of the latter include pericellular hyaluronan’s limiting the mobility of membrane-associated molecules [43] and hyaluronan’s forming macromolecular complexes with proteoglycans such as aggrecan and versican in the extracellular matrix [44]. With regard to timescales, thermal motions of the hyaluronan polymer can create dynamism in hyaluronan polymer conformation (“flexibility”) and non-covalent binding/unbinding can impart transience to hyaluronan complexes with proteins. Owing to these obstacles, atomic-resolution structural biology, both experimental and computational, has principally been focused on hyaluronan oligomers, as detailed in what follows. This has left as a largely unexplored frontier the confluence of atomic-level interactions that creates macromolecular complexes and the resulting emergent properties required for cell structure and signaling. An important example of this is the contrasting signaling characteristics of high molecular mass hyaluronan (>1000 kDa) vs. lower molecular mass hyaluronan (<200 kDa) [35].

## 2. Atomic-Resolution Experimental Structural Biology of Hyaluronan and Its Complexes

NMR spectroscopy is useful for atomic-resolution structural biology of flexible biomolecules, and enables their characterization in aqueous solution at ambient temperatures. In contrast, X-ray crystallography requires a well-ordered crystal of the biomolecule in which each unit cell has the same static arrangement of atoms; this precludes molecular flexibility and typically entails non-physiological solvent and very low temperatures. However, X-ray crystallography is capable of resolving atomic-resolution structures of small to very large biomolecules and biomolecular complexes, whereas NMR spectroscopy has historically been limited by biomolecule size, though recent advances are increasing this size limit [45,46]. The challenges to the study of hyaluronan by NMR spectroscopy or X-ray crystallography arising from size and flexibility are reflected in the Protein Data Bank (PDB) [47,48], which contains only 17 entries with hyaluronan, either by itself or complexed with various protein partners (Table 1).

Glycosidic dihedral angle values for hyaluronan coordinates in these entries are generally consistent with each other and are relatively limited in range (Table 2). The average (*ϕ*_14_, *ψ*_14_) and (*ϕ*_13_, *ψ*_13_) values are (−73.7°, 102.0°) and (−73.8°, −118.4°) and the associated standard deviations are (30.1°, 43.5°) and (26.4°, 44.9°). The structure 6X3M appears to be an outlier in this data set, and there are multiple instances of non-^4^C_1_ hyaluronan ring pucker geometries in that PDB entry. Excluding 6X3M from the analysis results in (*ϕ*_14_, *ψ*_14_) and (*ϕ*_13_, *ψ*_13_) averages of (−77.2°, 111.3°) and (−75.7°, −119.9°) and associated standard deviations of (11.7°, 22.7°) and (12.4°, 35.0°). Notably, the standard deviation in *ϕ*_14_ values is reduced by nearly a factor of three when 6X3M is excluded.

The earliest example in the PDB dates to 1975 and comes from fiber diffraction studies of hyaluronan sodium salts (PDB ID 3HYA) [49], which do not reflect the aqueous environments typical of physiological hyaluronan. The next chronological example, dating to 1994, is from solution NMR and is of the 3-mer GlcAβ1-3GlcNAcβ1-4(1-deoxy)GlcA (1HUA) [37]. Dating to 2001 is a crystal structure of the 2-mer GlcAβ1-3GlcNAcβ1 in a non-covalent complex with the Flavobacterium chondroitin AC lyase protein (1HM3) [51]. These latter two examples, one from solution NMR and the other from X-ray crystallography, illustrate the challenges associated with experimental atomic-resolution structural biology on hyaluronan: the NMR data were from a hyaluronan 8-mer (i.e., four disaccharide repeats) and the X-ray data were from a hyaluronan 4-mer, yet the respective entries deposited in the PDB were for a 3-mer and a 2-mer. In the case of the NMR study, all β1-3 linkages in the 8-mer were considered to be equivalent, and likewise with the β1-4 linkages, and therefore the resulting model for the full 8-mer can be produced by simply repeating the 3-mer conformation, and would not capture the actual conformational heterogeneity of the 8-mer or longer hyaluronan polymers. For the X-ray study, electron density was visible for only two of the four sugars in the 4-mer due to disorder, which demonstrates the inability of the methodology to handle flexible, conformationally heterogenous molecules. In both cases, the hyaluronan polymers studied are ~1000× shorter than the 1000–8000 kDa counterparts found in normal biological fluids.

**Figure 2:**
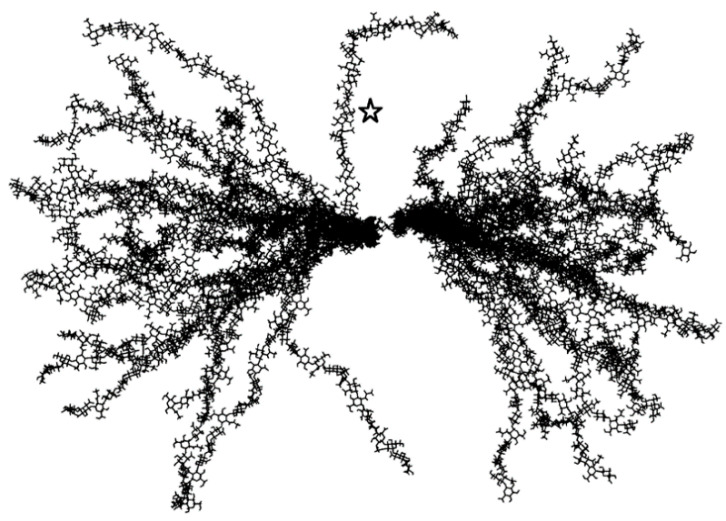
Conformational ensemble of hyaluronan 50-mers created by extrapolating from glycosidic linkage conformations sampled in molecular dynamics simulations of hyaluronan oligosaccharides. The star indicates a continuous linear stretch in one of the 50-mers. Reprinted with permission from Ref. [55]. Copyright 2006 Elsevier.

The second and only other solution NMR structure of hyaluronan in the PDB is from a 2006 study by Almond, DeAngelis, and Blundell [55]. While the deposited PDB structure (2BVK) contains only a single 8-mer conformer representing an average conformation, the authors discuss at length the flexibility of glycosidic linkages and the transient nature of hydrogen bonds seen in their complementary atomic-resolution explicit-solvent molecular dynamics simulations of hyaluronan oligosaccharides. In these simulations, transient intramolecular hydrogen bonding, in contrast to what is seen in fiber diffraction (3HYA), precludes stabilization of the glycosidic linkages and the acetamido groups, and there is substantial diversity in the ensemble of conformations sampled during the simulations. Extrapolating these findings to a ~10 kDa 50-mer gives a picture of a polymer capable of dramatic flexibility (Figure 2), even though it is still 100× or more smaller than hyaluronan polymers occurring in biology.

## 3. Atomic-Resolution Molecular Dynamics Simulations of Hyaluronan and Its Complexes

The importance of atomic-resolution explicit-solvent molecular dynamics simulations in complementing the NMR experiments associated with the 2BVK hyaluronan 8-mer structure, as described above, underlines the utility of such simulations. These simulations progress the conceptualization of hyaluronan away from having a particular three-dimensional conformation and toward having an ensemble of conformations. As such, this type of simulation has become increasingly common in characterizing the atomic-resolution conformational properties of hyaluronan in the context of various atomic cations and interacting with proteins and with lipids under physiological conditions. The number of publications describing these simulations currently exceeds the number of publications associated with the PDB entries in Table 1 by an order of magnitude.

### 3.1. Molecular Dynamics Simulation Methodology

To be clear, the phrase “atomic-resolution explicit-solvent molecular dynamics simulation” refers to the combination of an atomic-resolution force field model with an algorithm for integrating the equations of motion and thereby propagating the system through time. This has proven to be a powerful approach for generating conformational ensembles whose properties can be related to experimental observables [63,64] and that can provide free-energy estimates, through the application of statistical mechanics, for processes such as conformational transitions [65,66,67,68,69] and non-covalent binding/unbinding [70]. More commonly, a molecular dynamics trajectory is used directly as a “computational microscope” [71] to provide an atomic-resolution view of biomolecules that is not directly accessible by experimental methods.

Refinements to the force field models that describe interactions between atoms and advances in simulation software capable of effectively utilizing specialized computing hardware [72,73,74,75,76,77] have enabled increasingly longer and more accurate simulations on larger and larger systems of molecules. Atomic-resolution force field models widely used for carbohydrate simulations to-date represent each non-hydrogen atom as a single interaction site. Polar hydrogen atoms, for example in hydroxyl or acetamido groups, are also each represented by a single interaction site, while non-polar hydrogen atoms may or may not be implicitly combined with the carbon atom to which they are attached. Interestingly, this comparatively simple force field approach provides better descriptions of monosaccharide puckering, disaccharide 3D-conformation, and solvent interactions than substantially more complex semi-empirical quantum mechanics models in molecular dynamics simulations [78], which perhaps reflects the fact that high-level ab initio quantum mechanics computations, along with experimental NMR and crystallographic data, are routinely used in the development of force field parameters. The CHARMM [79,80,81], GLYCAM [82,83], and GROMOS [84,85,86,87,88] force fields are currently the most widely used atomic-resolution force field models for molecular dynamics simulations of carbohydrates, and have all undergone substantial development and validation efforts towards maximizing accuracy while also providing a diverse set of parameters that includes coverage for hyaluronan.

Numerical integration schemes have evolved to enable the generation of thermodynamically correct molecular dynamics trajectories under conditions of constant temperature and constant pressure, as appropriate for typical biological systems, and are now considered standard [89]. This has been an important advance, since simply applying Newton’s equations of motion yields a trajectory in which energy and volume are constant and temperature and pressure may be subject to undesirable fluctuations. Two additional relevant methodological advances are the introduction of Ewald summation to account for long-range electrostatic interactions [90], and the development of accurate parameters for monoatomic cations [91] since seemingly small changes to ion parameters can have large effects on the observed conformational properties of simulated glycosaminoglycans [92]. These latter two advances are especially relevant for hyaluronan simulations because of the net −1 charge that each hyaluronan disaccharide carries at near neutral pH on account of the carboxylic acid moiety with a low pKa (Figure 1). Given the long-range nature of charge-charge interactions and the large amount of negative charge on hyaluronan, errors in the treatment of electrostatic interactions along with a lack of or poorly parametrized counterions would be expected to have strong impacts on simulation results. Simulation studies combining atomic-resolution force fields with implicit or continuum solvent models cannot provide molecular details of water or ion interactions with hyaluronan and are therefore not included in this review.

Outside the scope of this review are polarizable force field models. Polarizable force field models do provide an atomic-resolution view of biomolecules, and additionally include electronic polarization effects dynamically during the course of a simulation, unlike classical force field models mentioned above [93,94,95,96,97]. Thus, polarizable force field models have the potential to be more accurate and more broadly applicable. However, such models are relatively new compared to classical fixed-charge force field models like CHARMM, GLYCAM, and GROMOS, and as such have not been widely used for carbohydrate simulations. There are only two examples, both published within the past three years, of molecular dynamics simulation of hyaluronan using a polarizable force field model [98,99]. Depending on the specific implementation, the inclusion of electronic polarization may also incur substantial additional computational cost, which reduces the timescales attainable in polarizable force field simulations relative to classical fixed-charge force field simulations.

Also outside the scope of this review are coarse-grained force field models; such models have been specifically developed for glycosaminoglycans [100], including hyaluronan [101,102,103,104]. Coarse graining condenses clusters of non-hydrogen atoms to a single interaction site, and therefore is not an atomic-resolution representation. However, this does have the advantage of reducing the associated computational costs relative to an atomic-resolution representation. Coarse-grained models may also use an implicit or continuum model of the water environment [101], which can substantially reduce computational costs relative to an explicit representation, and this can be especially true for simulations of extended polymers in which most of the system would otherwise consist of interaction sites representing water [102]. While it is not possible to directly observe the molecular details of water interactions using implicit or continuum solvent models, such models can nonetheless be useful for computing system properties such as osmotic pressure [105]. Alternatively, at the expense of substantial additional computational cost for dilute systems, coarse-grained models can include explicit water modeled as clusters of water molecules (that can optionally include polarizability) [106,107], which then enables direct investigation of ion effects on hyaluronan chains through the inclusion of explicit ions [104].

### 3.2. Molecular Dynamics Simulations of Aqueous Hyaluronan and Its Interactions with Itself, Other Glycosaminoglycans, and Ions

Early aqueous simulations of hyaluronan, some of which date back over three decades, were chiefly concerned with understanding its conformational properties and its intermolecular interactions with other glycosaminoglycan molecules. An important goal was to complement NMR and electron microscopy data to understand how oligosaccharide conformations implied by NMR and directly observed in the simulations contribute to the high polymer scale view provided by electron microscopy. In that early work, it was concluded that aqueous hyaluronan had a 2-fold helical structure that could support its non-covalent association with other hyaluronan molecules and with chondroitin [108,109]. As these simulations occurred early on during the development of molecular dynamics methods for biomolecules, they did not account for long-range electrostatic interactions nor did they include counterions. Furthermore, the timescales of the simulations were five to six orders of magnitude shorter than the timescales typical for similarly sized systems in the present day.

An important challenge to conformational characterization of hyaluronan by NMR is that of strong coupling and overlapping resonances [37,110]. In combination with 15N-NMR [55,110,111], molecular dynamics simulations have been helpful in addressing this challenge, leading to the conclusion that the local average structure of hyaluronan in solution is a contracted left-handed 4-fold helix [55], as opposed to the 2-fold helix from prior studies detailed above. Interestingly, this 4-fold average solution structure is very similar to that from X-ray fiber diffraction [49], despite transient hydrogen bonds that exchange with water in contrast to the water-depleted solid-state fibers. Importantly, these more recent simulations included both Ewald summation and explicit sodium ions. Expansion on this line of work entailed including residual dipolar couplings as experimental restraints and their application to select conformations from molecular dynamics simulation [38]. The simulations were of an aqueous hyaluronan 10-mer with explicit neutralizing sodium ions and Ewald summation of electrostatics. While this enhanced approach confirmed the prior 4-fold geometry, it also determined that the 4-fold geometry could interconvert to a 3-fold left-handed helix, which in fact was found to be more probable than the 4-fold geometry [38].

In addition to addressing the average conformation of oligomeric hyaluronan in aqueous solution and highlighting the presence of dynamic conformational fluctuations, molecular dynamics simulations have been used to better understand hyaluronan-water interactions at atomic resolution. The inclusion of explicit water molecules in the simulations was a critical ingredient enabling these structural insights. While direct inter-residue hydrogen bonding has the capacity to constrain rotation about glycosidic linkages, water-mediated hydrogen bonds can also do the same [112]. Water molecules can both break intramolecular hydrogen bonds in hyaluronan while also forming a water cage that paradoxically constrains glycosidic linkages [113]. Furthermore, the dynamic exchange of intramolecular hydrogen bonding facilitated by water molecules enables rapid conversion between low energy conformations of hyaluronan [114], which may explain the solubility of the flexible hyaluronan molecule even at large molecular masses, in contrast to other more rigid glycans that prefer to exist in the solid state [115].

Direct atomic-resolution explicit-solvent simulations of longer hyaluronan chains include a 40-mer constructed by multiplying by five the coordinates of the 4-fold left-handed helical 8-mer structure (Table 1, 2BVK) [116]. After a brief heating and equilibration phase, this construct was noted to maintain its helical conformation during a 10-nanosecond (-ns) simulation, in contrast to hyaluronan derivatives that were 6-sulfated at either every other or every fourth GlcNAc and that lost the helical starting conformation. The simulation was repeated but with CHCl_3_ replacing the water, and the hyaluronan 40-mer again maintained its helical conformation, demonstrating that, at least at the 10-ns timescale, this conformation is stable regardless of the polarity of the solution, which is consistent with this conformation also being observed in the solid state [49,55].

**Figure 3:**
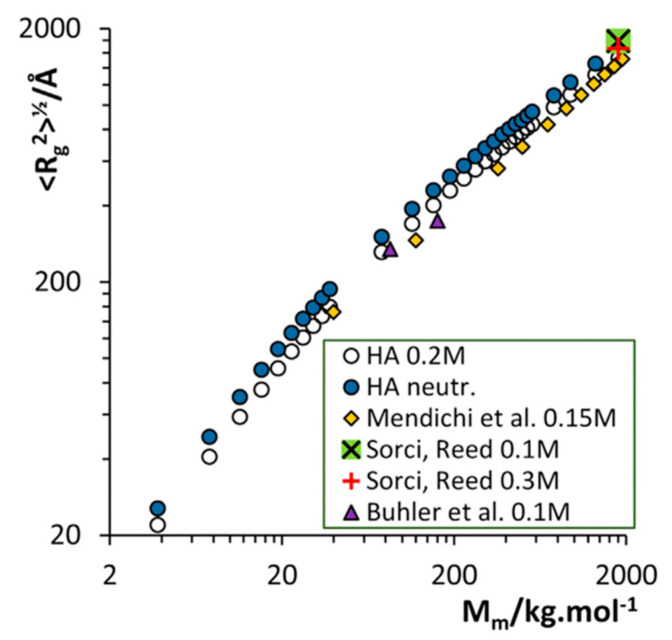
Radii of gyration (R_g_) of modeled hyaluronan random coils of varying molecular mass (M_m_) constructed using data from molecular dynamics simulations with either 0.2 M NaCl (white circles) or neutralizing sodium (blue circles) as compared to various experimental data (other symbols; see [118] for references to experimental data). Reprinted with permission from Ref. [118]. Copyright 2017 Elsevier.

All of these simulations were on hyaluronan oligosaccharides, with the 40-mer simulations being the longest oligomer in the series of published simulations summarized above. These obviously do not directly address the question of the behavior of longer hyaluronan polymers. To this end, investigators have utilized conformational data from their oligomer simulations to extrapolate to longer lengths. It was noted that in 4-mer simulations the peripheral glycosidic linkages sampled secondary minima, from which it was hypothesized that long hyaluronan polymers could have folds, loops, and turns if they sampled such linkage conformations [117]. The results of subsequent 8-mer simulations were used to construct an ensemble of 50-mer conformations by randomly selecting glycosidic linkage conformations from the simulation data [55]. More recently, 100-ns timescale molecular dynamics simulations of hyaluronan 48-mers under varying concentrations of NaCl and MgCl_2_ were used to extrapolate to higher molecular mass structures containing up to 10,000 monosaccharides (Figure 1, *n* ≤ 5000; molecular mass ≤ ~2000 kDa) [118]. Randomly selected pieces of hyaluronan from the simulation trajectories were connected using glycosidic linkage dihedral angles as occurring in the simulations, similar to previously described construction approaches that used glycosidic conformational probabilities as determined by estimates of conformational energies [119,120]. This construction approach, which assumes independence of the conformational probabilities of different glycosidic linkages in the polymer, was very successful at generating hyaluronan conformations whose radii of gyration closely matched experimental values at different NaCl solution concentrations as well as a large range of polymer lengths [118] (Figure 3).

The independence of the conformational probabilities of different glycosidic linkages in a hyaluronan polymer has indeed been confirmed in microsecond-timescale atomic-resolution explicit-solvent molecular dynamics simulations of hyaluronan 10- and 20-mers [121]. In using independent conformational probabilities for each linkage, it is possible when constructing the polymer to generate nonphysical chain conformations having intramolecular clashes that contain overlapping bonds or having a bond piercing the center of a monosaccharide ring. However, these nonphysical chain conformations are very rare and can be readily detected for exclusion by the application of a brief energy minimization combined with a maximum bond potential energy criterion [121,122].

**Figure 4:**
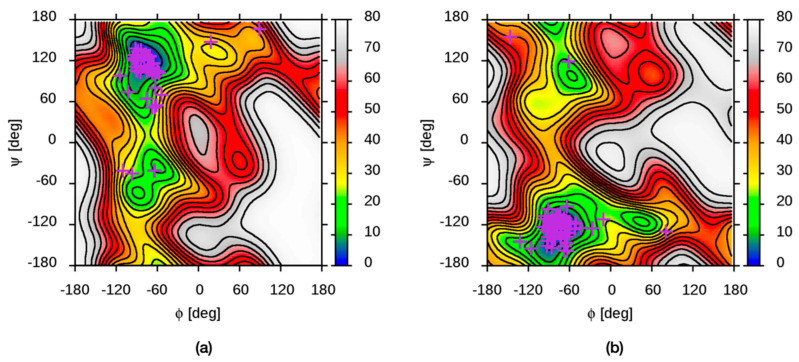
Computed hyaluronan disaccharide conformational free energies compared with all available PDB hyaluronan structural data for (**a**) (*ϕ*_14_, *ψ*_14_) and (**b**) (*ϕ*_13_, *ψ*_13_) glycosidic linkage dihedrals. Free energy data, in kJ/mol and shown as contours, are adapted with permission from Figure S1 from reference [123], copyright 2021 American Chemical Society, and PDB data, shown as +’s, are from Table 2 in the present work. Free energies were computed from all-atom explicit-solvent molecular dynamics simulations of (**a**) GlcNAcβ1-4GlcA and (**b**) GlcAβ1-3GlcNAc disaccharides using the CHARMM force field.

Enhanced sampling molecular dynamics simulations on hyaluronan disaccharides provide comprehensive free energy values as a function of glycosidic linkage dihedral angles, including for values rarely sampled in conventional (unbiased) simulations [123]. Locations of free-energy minima from these disaccharide simulations compare favorably with conformational data from hyaluronan structures in the PDB (Figure 4). These conformational free energies can also be used for polymer construction, with the caveat that the conformational properties of the glycosidic linkage in a disaccharide may be somewhat different than the same linkage in the middle of a longer oligomer due to end effects [117].

**Figure 5:**
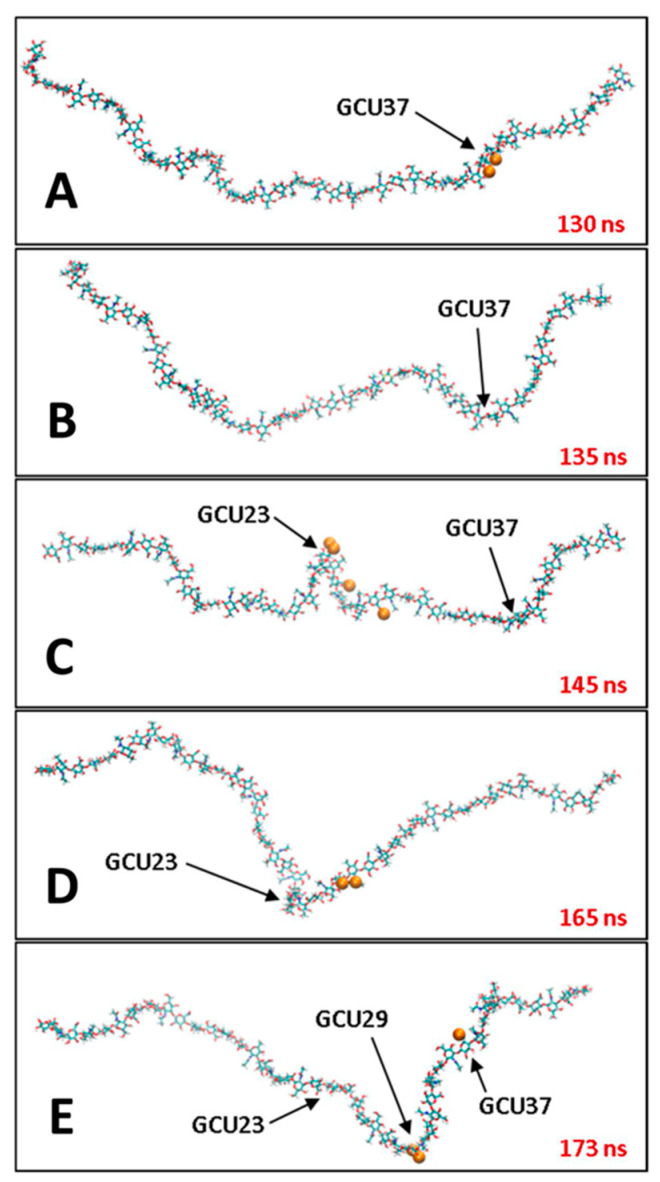
Hairpin-like turns induced by Na^+^ binding to hyaluronan in atomic-resolution explicit-solvent molecular dynamics simulation. Panels (**A**–**E**) are successive frames from a single molecular dynamics trajectory demonstrating that Na^+^ binding either immediately precedes or coincides with turn formation. Na^+^ ions are drawn as orange spheres, and arrows point to the glucuronate residues where the events occur (“GCU37”, “GCU23”, “GCU29”). The elapsed simulation time, in nanoseconds, is in red font. Reprinted with permission from Ref. [126]. Copyright 2020 Elsevier.

An important finding from simulations with explicit ions is that the hyaluronan polymer chain becomes less stiff with increasing cation concentration, and that, in comparing simulations with Na^+^ vs. with Mg^2+^, this effect is independent of the cation type despite important differences in the atomic-resolution details of the hyaluronan-cation interactions [118]. Specifically, Na^+^ favors direct contact with the polymer while Mg^2+^ prefers interactions bridged by water molecules; the electrostatic shielding effect of the latter type of interaction may explain why the change in chain stiffness, as measured by radius of gyration, is independent of the ion type and depends only on its concentration [118]. The decrease in hyaluronan stiffness has also been seen in atomic-resolution explicit-solvent molecular dynamics simulations of hyaluronan with Ca^2+^ corroborated with single-chain force spectroscopy [124]. In both the Na^+^/Mg^2+^ study and the Ca^2+^ study, cation interaction with the negatively charged carboxylate group was observed [118,124]. In the case of Na^+^, radial distribution functions demonstrated its binding to the carboxylate on GlcA residues to be much more favorable than to hydroxyl groups [125]. Detailed solvation shell analysis revealed specific interaction points that could be occupied by Na^+^, which induce glycosidic dihedral conformational change that cause hairpin-like turns that compact the chain [126] (Figure 5). Finally, while Na^+^ is more likely to form direct interactions with hyaluronan than is Mg^2+^, as discussed above, K^+^ is even more likely than Na^+^ to form direct interactions [127]. This arises from the larger ionic radius of K^+^ compared to Na^+^ that, combined with polymer rigidity limitations, makes direct K^+^ bridging of carboxylate groups more likely than solvent-separated K^+^ bridging [127].

There have been a limited number of atomistic explicit-solvent molecular dynamics simulations in which the simulation systems contained more than one molecule of hyaluronan or a combination of hyaluronan and another glycosaminoglycan. Such systems have served as simplified models aimed at providing an atomic-resolution view of the physical properties of biological tissues. Toward better understanding the lubricating characteristics of glycosaminoglycans with regard to articular cartilage, it was observed that in a 10-ns simulation of a dense system containing a single long hyaluronan molecule with a single chondroitin sulfate molecule, both of which had multiple bends and kinks, there occurred intermolecular hydrogen bonds that lasted as long as the full 10 ns [128]. As expected, repeating the simulation at 300, 310, and 320 K resulted in shorter-duration hydrogen bonding at higher temperature [128]. Two fully extended molecules of hyaluronan measuring 30 nm each were placed parallel to each other and subjected to tensile and to compressive loading to investigate the nanomechanics of intervertebral disk annulus fibrosus extra-fibrillar matrix [129]. Under tensile loading, water molecules debonded from the hyaluronan molecules whereas under compressive loading, the hyaluronan coiled extensively and thereby trapped more water molecules through hydrogen bonding interactions, which could help to sustain compressive loads [129]. As a simplified representation of pericellular glycocalyx, four hyaluronan 32-mers in linear extended conformations measuring 16 nm were oriented parallel to each other along the long axis of a simulation box of dimensions 4 nm × 4 nm × 48 nm and restrained in place; the atomic-resolution simulation results from this system identified emergent properties, including reversal of the Donnan potential, which can be useful for mean-field modeling [99]. Two antiparallel hyaluronan 48-mers were simulated at 275 K and 310 K and in varying concentrations of NaCl up to 1 M to investigate the possibility of duplex formation [126]. The hydrophilic nature of hyaluronan combined with intermolecular electrostatic repulsion from the carboxylate groups disfavors association of the two strands. With the addition of NaCl up to 0.6 M, electrostatic screening reduces the effect of electrostatic repulsion, and close approach between the two strands becomes somewhat more probable. However, this trend inverts at higher NaCl concentration as hydrogen bonding becomes successively weaker. Ultimately, the investigators concluded that, in aqueous solution, hyaluronan duplex structures are not stable [130].

This review focuses on molecular dynamics simulations employing classical atomic-resolution force-fields as this type of simulation accounts for the vast majority of published atomic-resolution simulations using explicit solvent. With these force fields, no covalent bond breaking or formation is possible, and therefore the studies are limited to investigating conformational changes and non-covalent association/dissociation events. There do exist a limited number of studies using reactive molecular dynamics, in which covalent bond reactions are possible, to study bond breaking in hyaluronan. These have focused on rupture of hyaluronan as relevant for shock-induced damage of perineuronal nets as occurs in traumatic brain injury [131,132].

### 3.3. Molecular Dynamics Simulations of Hyaluronan–Protein Complexes

Atomic-resolution explicit-solvent molecular dynamics simulations of hyaluronan–protein complexes fall into two broad categories. The first is simulations based on existing atomic-resolution experimental structures of hyaluronan–protein complexes. This first category is quite small and is limited to complexes of hyaluronan oligosaccharides either with bacterial lyase or with the hyaluronan-binding domain of the CD44 transmembrane protein, as detailed in Table 1. The second is simulations of hyaluronan–protein complexes that fall outside of this category.

*Streptococcus pneumoniae* hyaluronan lyase degrades hyaluronan in the extracellular matrix of host organisms, and thereby contributes to bacterial pathogenicity. 50-ns molecular dynamics trajectories of *S. pneumoniae* hyaluronan lyase were used to confirm three types of previously described large-scale motions that confer flexibility to the protein relevant to its enzymatic function [52,53,133]. Principal component analysis of snapshots from trajectories of apo and holo structures showed that two of the three motions were largely independent of the presence of bound hyaluronan oligosaccharide, whereas opening/closing of the substrate-binding cleft became more limited when hyaluronan is bound, that is, in the holo form of the enzyme [133]. Additional sub-microsecond length simulations were used to put forward a processive mechanism for exolytic hyaluronan degradation by the enzyme that was dependent upon the three large-scale protein motions [134]. The actual chemistry of the degradation mechanism was subsequently investigated with molecular dynamics simulations and a quantum mechanics/molecular mechanics approach, leading to proposal of a mechanism for the complete catalytic cycle [135,136].

CD44 is a transmembrane protein involved in the biology of human cancers and contains an extracellular hyaluronan-binding domain that allows it to mechanically tether the cell membrane to pericellular hyaluronan and to transduce signals into the cell based on this binding. The hyaluronan-binding domain has been crystallized in both an “A-form” and a “B-form”, which both have hyaluronan oligosaccharide bound in the same pose, but have distinct conformations for an arginine side chain that either is separated from the bound oligosaccharide in the A-form structure or makes direct contact in the B-form structure [56]. In one set of molecular dynamics simulations on the complex, changing the peptide backbone conformation of an tyrosine residue adjacent to the arginine was shown to drive conformational switching between the A- and B-forms, which were otherwise stable [137]. In contrast, in a separate simulation study the A- and B-forms were not observed as separate stable states due to the arginine sidechain being in rapid conformational flux independent of the tyrosine backbone [138]. A possible explanation for this is that the two studies used different force fields, and this result highlights the importance of force field accuracy to molecular dynamics simulation results. Additional simulations on the complex demonstrated that hyaluronan must shed water to form the bound complex and that water molecules that are retained in the binding interface form a thin rigid layer [139,140]. Separate from the binding-site arginine conformational change is an order-to-disorder transition in a group of amino acids far removed from the crystallographic hyaluronan binding site that nonetheless contributes to binding. From simulation of the initial stages of this order-to-disorder transition, a tyrosine residue in that region experimentally determined to be important to the order-to-disorder transition was surprisingly found not to contribute to the stability of the ordered state via the anticipated local interactions during the early steps of the transition [141]. Simulations of the disordered state revealed a likely molecular mechanism by which the order-to-disorder transition increases the binding affinity for hyaluronan: positively-charged amino acids distant from the hyaluronan binding site in the ordered state gain sufficient mobility in the disordered state to directly interact with bound hyaluronan [142]. Furthermore, force from the C-terminus can disrupt the ordered structure and induce the transition to the disordered state, which is relevant to affinity switching of the CD44 receptor under conditions of shear stress, as occurs during cell trafficking events [143]. Results of extensive unbiased molecular dynamics simulations led to the proposal of binding modes in addition to the crystallographic binding mode that may be important to the initial stages of binding [144]. Docking studies resulted in two binding modes similar to those seen in the prior study, and these docked conformations were then used for molecular dynamics simulations to study the coupling of binding affinity to the order-to-disorder transition, which suggested that a folding of the disordered state to a compact conformation results in an increase in binding affinity [145]. Molecular dynamics simulations have also been used to study possible differences in the molecular details of hyaluronan binding to different splice variants of CD44, which were constructed by homology modeling [146]. Finally, simulations of glycosylated CD44 hyaluronan-binding domain revealed that terminal sialic acids on the attached glycans could interfere with hyaluronan binding by competing for interactions with arginine sidechains important for hyaluronan binding [147,148].

While molecular dynamics studies on hyaluronan–lyase and hyaluronan–CD44 complexes benefitted from existing atomic-resolution structural biology of the respective complexes, in a number of instances modeling was applied to extend the structural biology, for example to explore additional putative hyaluronan binding modes [144,145,146] or to add N-linked glycosylation to the protein partner [147]. Such modeling has been applied to a large number of other hyaluronan–protein systems, for which experimental atomic-resolution structural biology of the bound pose(s) does not exist. On the one hand, the results from such works ought to be interpreted with caution. On the other hand, those modeling efforts can enable atomic-resolution views of biology that would otherwise be unavailable. The remainder of this section contains a necessarily brief overview on the large number of these efforts, which are limited to the last decade—a time frame that coincides with the improvements in models, methods, and computing hardware that enable such work.

**Figure 6:**
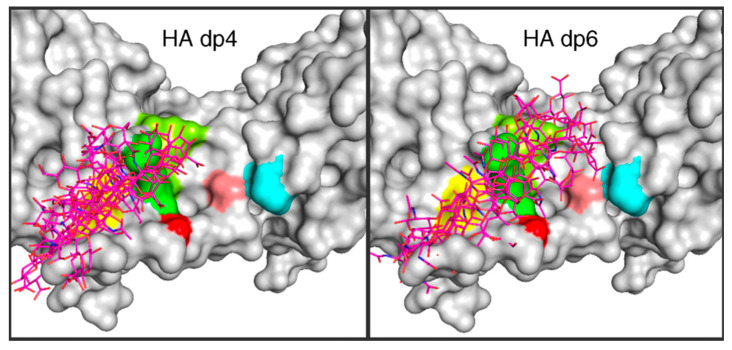
Ensemble of computationally predicted binding poses for hyaluronan 4-mers (“HA dp4”) and 6-mers (“HA dp6”) with IL-10. Dynamic Molecular Docking (DMD) [165] was used to generate the ensemble. DMD is an atomic-resolution explicit-solvent molecular dynamics method where full flexibility of both the protein and ligand are allowed throughout a molecular dynamics trajectory during which the ligand is gradually pulled toward the protein. Reprinted with permission from Ref. [154]. Copyright 2015 Elsevier.

There exist a substantial number of studies on hyaluronan–cytokine complexes that have employed atomic-resolution explicit-solvent molecular dynamics simulations. Many of these studies include the use of chemically sulfated hyaluronan, which, while distinct from the exclusively non-sulfated naturally occurring form, is a useful analogue of sulfated glycosaminoglycans in the context of biophysical and biochemical assay. In the case of interleukin-8, it was determined that (non-sulfated) hyaluronan is expected to bind in a pose common to sulfated hyaluronan, chondroitin sulfate, dermatan sulfate, and heparin, and that hyaluronan glycosidic linkage conformations do not significantly change in going from the unbound to the bound state [149]. Sulfated hyaluronan, as well as chondroitin sulfate, were used to probe glycosaminoglycan binding to bone morphogenetic protein-2 (BMP-2), leading to the conclusion that binding depends on both the degree and the pattern of glycosaminoglycan sulfation [150]. In contrast to sulfated hyaluronan, natural non-sulfated hyaluronan is not a good binding partner for BMP-2 at neutral pH, as BMP-2 carries a net -8 charge; however, at a pH of 4.5, due to protonation of titratable groups on the protein, the net charge on BMP-2 changes to +4, which enables binding with non-sulfated hyaluronan, whose carboxylic acid functional groups have a pKa of ~3 and are therefore largely in the negatively charged carboxylate form at both pH 7 and pH 4.5 [151]. From molecular dynamics simulations, hyaluronan oligosaccharide interaction with CXCL8, as well as with interleukin-10, heparin-binding EGF-like growth factor, CXCL14, and VEGF_165_, was determined to be weaker than for sulfated glycosaminoglycans including sulfated hyaluronan, and this was the same trend seen experimentally [152,153,154,155,156,157] (Figure 6). Sulfated hyaluronan was shown to have a clear binding pose in its complex with the TGF-β1/TβR-I/TβR-II complex that is energetically favorable and electrostatically driven, in contrast to non-sulfated hyaluronan, and a particular lysine residue in TβR-I is key to interactions with sulfated hyaluronan carboxylate and sulfate functional groups [158]. Similarly, non-sulfated hyaluronan oligosaccharide barely interacts with the complex of PDGF and its receptor PDGFR-β, whereas chemical sulfation of hyaluronan substantially increases its binding [159]. While directly getting estimates of binding free energies from standard atomic-resolution explicit-solvent molecular dynamics simulation is a difficult task, it is relatively easy to do so by applying a continuum solvent approximation to snapshots taken from explicit-solvent molecular dynamics trajectories using the molecular mechanics Poisson-Boltzman surface area (MM-PBSA) or molecular mechanics generalized Born surface area (MM-GBSA) methods [160,161,162,163], and these methods are useful tools in the context of glycosaminoglycan–protein binding [164], including as applied to the hyaluronan–cytokine complexes above.

Atomic-resolution explicit-solvent molecular dynamics have also been applied toward furthering the understanding of hyaluronan interaction with the protein albumin, as this pairing is believed to be important in the lubrication mechanism of synovial fluid. An important consideration for this pairing is that, under the relevant physiological conditions, both molecules carry a net fixed negative charge. Therefore, from purely the perspective of electrostatic interactions, it would be expected that the two molecules would be in a more favorable free energy state when dissociated from each other. This is in contrast to cytokines, which contain basic amino acids whose positively charged side chains can form electrostatically complementary interactions with glycosaminoglycans [166,167,168,169,170], as discussed above. While continuum descriptions of electrostatics can include electrostatic effects due to ions [171,172], they cannot model the atomic-resolution interactions between hyaluronan, albumin, water, and cations that contribute to charge compensation. In comparing the effects of Na^+^, Ca^2+^, and Mg^2+^ cations on stabilizing hyaluronan–albumin binding in molecular dynamics simulations, the two divalent cations were found to have a stronger stabilizing effect [173]. Of the two, Ca^2+^ had the stronger stabilization effect, with a greater tendency to form cation-mediated bridges between hyaluronan and albumin, presumably because of the lower hydration of Ca^2+^ relative to Mg^2+^ [173]. The greater capacity of Ca^2+^ to form ionic interactions with the two biomolecules as compared to either Na^+^ or Mg^2+^ has been corroborated by second set of independent simulations [174].

**Figure 7:**
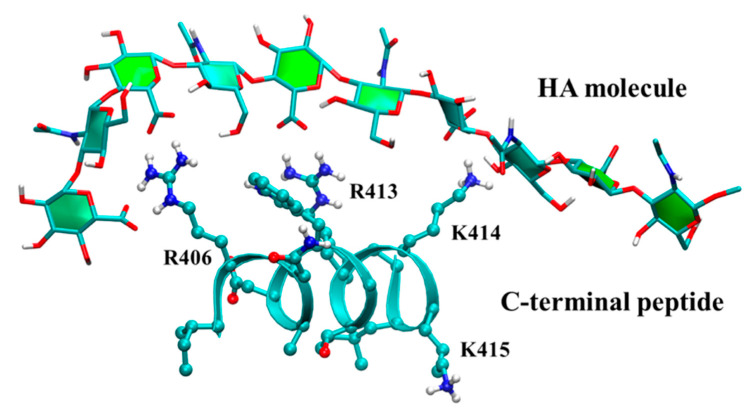
Molecular dynamics snapshot of a hyaluronan oligosaccharide interacting with the C-terminal region of *Streptococcus equisimilis* hyaluronan synthase. Probability densities computed from molecular dynamics snapshots show that R406 and R413 sidechains interact strongly with hyaluronan while the K414 sidechain interaction is weak. Reprinted with permission from reference [175], copyright 2017 American Chemical Society.

Molecular dynamics simulations have also been applied toward understanding the process of hyaluronan synthesis by hyaluronan synthases. Simulations detailed contacts between hyaluronan and arginine but not lysine residues in the C-terminal region of *Streptococcus equisimilis* hyaluronan synthase critical for enzyme function [175] (Figure 7). Cryo-electron microscopy structures of a viral hyaluronan synthase homologue were supplemented by computational addition of a hyaluronan 6-mer to the experimental structure in different registers and subsequent molecular dynamics simulation to discover significant register-dependent stability of hyaluronan, which suggests a mechanism for how transit of the carbohydrate polymer through the protein pore is coupled to elongation of the carbohydrate [176].

The prevalence of unique atomic-resolution explicit-solvent molecular dynamics simulations within the past five years has substantially broadened the investigation of hyaluronan–protein and hyaluronan–peptide interactions, and the remainder of this section contains a comprehensive and necessarily brief summary of these efforts. The mammalian glycoprotein YKL-40, whose physiological ligand was unknown, was shown to preferentially bind hyaluronan using specialized simulations that combined free energy perturbation and Hamiltonian replica exchange molecular dynamics [177]. MM-GBSA analysis of molecular dynamics trajectories seeded with various docked conformations of glycosaminoglycans showed that hyaluronan could simultaneously bridge both proteins in the MMP2/TIMP3 complex and stabilize the complex to increase MMP2 activity, unlike other glycosaminoglycans [178]. Two different forms of serum amyloid A protein, only one of which is found in amyloid deposits, were independently simulated with hyaluronan, and these microsecond-scale trajectories showed distinct hyaluronan binding patterns that may be important to the initial protein oligomerization leading to amyloid formation [179]. The polysaccharide lyase from the *Stenotrophomonas maltophilia* bacterium has pH-dependent activity against three chemically dissimilar carbohydrates, including hyaluronan, and simulation data directly correlated stability of hyaluronan–protein binding at various pH values with hyaluronan lyase activity of the enzyme as a function of pH [180]. Investigation of hyaluronan as a putative inhibitor of sucrase-isomaltase, an enzyme relevant for type 2 diabetes, demonstrated that hyaluronan can induce a conformational change in a stretch of amino acids required for normal catalytic function [181]. Small hyaluronan fragments were determined to be especially capable of decreasing the rate of water permeation through the transmembrane water channel aquaporin-3, which may be relevant to the mechanisms by which hyaluronan can moisturize skin [182]. Molecular dynamics simulations have been applied to compare the capacity of chitosan vs. hyaluronan to bind to collagen [183]. MM-GBSA post-processing of simulation trajectories of hyaluronan with five different cathepsin proteins showed a ~15 kcal/mol range in binding affinity based on the cathepsin type and the hyaluronan length, with the binding affinity showing either a direct, an inverse, or no correlation with hyaluronan length depending on the cathepsin type [184].

An 11 amino acid basic peptide from link protein was simulated with a hyaluronan 10-mer, resulting in electrostatically stabilized interactions, which were also seen with a peptide containing the same 11 amino acids but in a scrambled sequence but not in one where the five basic residues were replaced with glycine, all of which was consistent with experimental findings [185]. A 19 amino acid N-terminal peptide containing five arginine and two lysine residues from glial-cell-line-derived neurotrophic factor was tested for its ability to bind a series of glycosaminoglycans of varying sulfation [186]. In unbiased simulations of the glycosaminoglycan–peptide systems, three arginine residues formed hydrogen bonds across the series, but hyaluronan typically formed only two hydrogen bonds whereas sulfated glycosaminoglycans formed between five to seven hydrogen bonds [186].

### 3.4. Molecular Dynamics Simulations of Hyaluronan–Lipid Complexes

A number of published atomic-resolution explicit-solvent molecular dynamics studies characterize the interaction of hyaluronan with lipids as relevant for the lubricating properties of synovial fluid. For example, low molecular mass hyaluronan simulated with dipalmitoylphosphatidylcholine (DPPC) in aqueous solution can absorb into the phospholipid component, which prevents formation of supramolecular interactions important for lubrication [187]. Furthermore, while under normal conditions wherein hyaluronan is extended, the hyaluronan–phospholipid interactions do not compete with the lubricating hyaluronan–hyaluronan interactions, in contrast to pathophysiologic conditions in which hyaluronan molecules are coiled and phospholipid disrupts hyaluronan–hyaluronan interaction [188]. The phospholipids were found to have the most disruptive effect on hydrogen bonding networks between hyaluronan chains when the hyaluronan chains are small [189], and the existence of specific sites where hyaluronan hydrogen bonds with phospholipid was noted [190]. In the case where phospholipids form a micelle, it is possible that a hyaluronan chain can wrap around the micelle and that rolling and sliding motions of the micelle relative to the hyaluronan can lower friction in the system, thereby aiding lubrication [191]. When considering three different types of lipids, with each type accounting for 30% or more of the lipid content of synovial fluid, hyaluronan was found to hydrogen bond with each type for the same duration, in contrast to chondroitin sulfate, which, when 6-sulfated, could form hydrogen bonds of longer duration with one of the three lipid types [192]. When the phospholipid component takes the form of a bilayer, interaction between a hyaluronan polymer chain is stabilized by the presence of Ca^2+^ cations that, unlike Na^+^, form persistent ion bridges between hyaluronan carboxylate and lipid phosphate functional groups [193,194]. Furthermore, water-mediated interactions between lipid headgroups and hyaluronan, supported by redistribution of ions, are especially important in stabilizing the association of increasingly larger hyaluronan polymers with the bilayer [195].

### 3.5. Molecular Dynamics Simulations of Hyaluronan in the Context of Pharmaceuticals

Covalent conjugates of hyaluronan that contain a hydrophobic moiety can assemble into nanoparticles capable of carrying drug or imaging molecules, and simulation data on individual conjugate molecules have been correlated with experimental results in bulk solution [196]. This work was extended to encompass aromatic as well aliphatic hydrocarbons as the hydrophobic group: evaluation of the physicochemical properties of these conjugates not only in simulations of a lone molecule but also in systems containing either 22 molecules of the conjugate or in systems containing 4 molecules of the conjugate plus 2 molecules of a fluorescent dye provided an atomistic view of both hydrophobic and π-π stacking interactions as well as water exclusion in the supramolecular assemblies [197]. Whereas these studies focused on hyaluronan conjugates that were the sole ingredient of the nanoparticles, other work has investigated hyaluronan conjugates that can assemble onto liposome particles that in turn can carry an active pharmaceutical ingredient. Hyaluronan–phosphoethanolamine covalent conjugates can decorate the surface of a liposomal bilayer through incorporation of the phospholipid component of the conjugate into the bilayer, but this binding can affect the deformability of the bilayer, and simulations have been employed to better understand how different phosphoethanolamines have differing such effects [198]. Atomic-resolution explicit-solvent molecular dynamics simulations have also been used to rationalize the efficiency of the synthesis of conjugates of hyaluronan with a hydrophobic functional group as a function of solvent conditions containing a mixture of water plus an organic solvent. Specifically, having tert-butanol vs. 1,4-dioxane as the organic component of the mixed water-organic solvent results in substantial differences in the solvation shell surrounding hyaluronan, including much more enrichment of the solvation shell by water molecules in the case of tert-butanol [199].

Hyaluronan polymers have been studied as stabilizing agents or as matrix depots for protein pharmaceuticals. Hyaluronan was compared to chitosan, alginate, cyclodextrin, and pectin with regard to its ability to stabilize erythropoietin from thermal denaturation and was determined not to be the best option in the series [200]. A similar conclusion was drawn in the case of interleukin-2 protein [201]. MM-PBSA calculations using molecular dynamics trajectories of BMP-2 with protonation states appropriate for acidic and for neutral pH showed that, under acidic conditions, electrostatic interactions become an important contributor for its association with hyaluronan, which provides a molecular level explanation for differing sequestration and release properties of BMP-2 from hyaluronan in a hydrogel under these differing pH conditions [151]. Sulfation of hyaluronan was determined to increase binding to heparin-binding EGF-like growth factor protein relative to natural non-sulfated hyaluronan, which is relevant to the development of hyaluronan-containing hydrogels for controlled delivery of the protein in pharmaceutical applications [155]. Polyelectrolyte multilayers, composed for example of negatively charged hyaluronan and positively charged poly-L-lysine, have utility in drug delivery applications. Molecular dynamics simulations were used to directly characterize the assembly properties of this polyelectrolyte system onto a lipid membrane as a function of temperature, salt concentration, and poly-L-lysine size [202].

Hyaluronan can be utilized as a component of hydrogel or dissolving microneedles that are used in microneedle patches to painlessly deliver pharmaceutical compounds through the external stratum corneum layer of the skin and into deeper layers. The interaction preferences and details of hyaluronan and of polyvinyl alcohol, both of which are used to fabricate microneedles, with sulfonhodamine B, which was used as a model small molecule pharmaceutical compound, were determined using explicit-solvent molecular dynamics simulations [203]. Similar efforts were applied to evaluate hyaluronan vs. polyvinyl alcohol interactions with insulin, and, as was the case with sulfonhodamine B, the interaction with hyaluronan was strongly preferred [204]. Methacrylated hyaluronan, which can be photo-cross-linked for use in microneedle patches, was found to interact strongly enough with a somatostatin receptor type 2 antagonist peptide to enhance the peptide’s stability and rigidity relative to aqueous solution in the absence of methacrylated hyaluronan [205].

The above examples demonstrate the utility of atomic-resolution explicit-solvent molecular dynamics simulations toward understanding the behavior of hyaluronan as a component of nanoparticles, of matrix depots, and of microneedles, as well as a stabilizer for protein therapeutics. In addition to these applications wherein hyaluronan is part of the formulation for non-hyaluronan active pharmaceutical ingredients, hyaluronan has been simulated in contexts in which it was either covalently conjugated to an active pharmaceutical ingredient or a candidate active pharmaceutical ingredient itself. Hyaluronan was covalently conjugated to the antidiabetic pharmaceutical metformin and the non-covalent interaction of functional groups on the conjugate with functional groups on a phospholipid was determined through simulations as part of a combined experimental-simulation study investigating the use of CD44-targeted metformin for pancreatic cancer [206]. Hyaluronan was also evaluated for its ability to inhibit directly the SARS-CoV-2 M^pro^ protease for the purpose of developing therapy for this virus that is the cause of the COVID-19 pandemic, and docking studies rated hyaluronan as the top hit from a set of 2454 approved drug molecules with subsequent molecular dynamics simulations demonstrating stability of the predicted hyaluronan-M^pro^ complex up to 500 ns of simulation time [207].

## 4. Conclusions and Outlook

X-ray crystallography and NMR studies have laid the foundation for understanding the atomic-resolution structural biology of hyaluronan. Critical contributions from NMR have included the conformational properties of hyaluronan oligosaccharides in solution, including development of the view that hyaluronan is a biopolymer with intrinsic flexibility but also with clear preferences for certain ranges of values for glycosidic linkage dihedral angles. X-ray diffraction studies have provided a window into hyaluronan oligomers interacting with protein binding partners. Explicit-solvent molecular dynamics simulations have built on this foundation to provide atomic-resolution views of hyaluronan interactions with other hyaluronan and glycosaminoglycan molecules, with various monoatomic ions, with proteins and peptides, with lipids, and with drugs and drug-like molecules.

**Figure 8:**
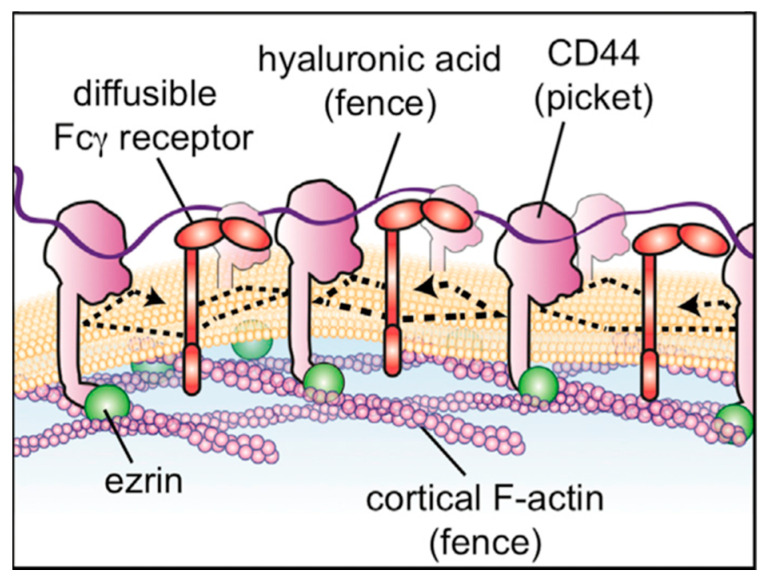
”Picket fence” on the cell membrane created by a single molecule of extracellular hyaluronan participating in polyvalent binding with multiple transmembrane CD44 molecules. Reprinted with permission from Ref. [43]. Copyright 2018 Elsevier.

A major task that remains is to computationally build and simulate larger hyaluronan-containing systems analogous to those occurring in biology. One such system is the pericellular hyaluronan coat anchored to the cell membrane via CD44 [43]. Challenges to this include: modeling the transmembrane region of CD44 embedded within a lipid bilayer; accounting for the order-to-disorder transition in the membrane-proximal extracellular portion of CD44; properly positioning multiple CD44 molecules in binding poses along the length of a single long hyaluronan polymer; and properly positioning the intracellular segments of multiple CD44 molecules in relation to intracellular protein binding partners (Figure 8). Another such system is the macromolecular complex formed by hyaluronan with proteoglycans in the extracellular matrix [44]. A challenge common to this and the CD44-containing membrane system is the positioning of multiple hyaluronan binding proteins along the length of a single long hyaluronan polymer. Unique challenges include construction of intact proteoglycans, which have protein portions that are intrinsically disordered and have covalently attached non-hyaluronan glycosaminoglycans, and proper accounting for ionic interactions between monovalent cations in solution and the massive amount of fixed negative charge owing to the carboxylate functional groups on hyaluronan combined with the carboxylate and sulfate functional groups on the non-hyaluronan glycosaminoglycans. While the challenges are large, atomic-resolution molecular dynamics simulations of these systems can enable the direct observation of the effects that hyaluronan association has on the biophysics of the cell membrane, of proteins associated with the cell membrane, and of proteins, glycosaminoglycans, and ions in the extracellular matrix at a resolution unattainable by experimental methods for such complex, heterogeneous, and dynamic systems.

While atomic-resolution explicit-solvent molecular dynamics simulations have enabled investigation of hyaluronan polymers longer than in the experimental studies reviewed here, and may, with the appropriate use of supercomputing resources, be applied to the CD44- or proteoglycan-containing systems proposed in the previous paragraph, the length of polymers in such simulations is still two orders of magnitude smaller than physiological hyaluronan. A major barrier to progress is that every 10-fold increase in hyaluronan length results in a 1000-fold increase in the size of the system to be simulated because the linear increase in the polymer length needs to be accommodated in all three dimensions by the simulation system. As such, direct atomic-resolution explicit-solvent molecular dynamics simulation of hyaluronan of physiological sizes, that is, 1000–8000 kDa, continues to be computationally infeasible despite the massive increase in computing power over the past three decades since the earliest such simulations were performed. Therefore, for the foreseeable future, progress toward simulation of successively larger and more realistic hyaluronan-containing systems will likely be achieved by a combination of experiments and multiscale simulation approaches. Classical fixed-charge force field studies as reviewed in this article can be used to develop, refine, and validate coarse-grained models, which in turn enable treatment of much larger systems that more closely mimic the composition of biological systems in terms of size. Polarizable force field models, which are more complex than fixed-charge force fields, may prove to be important in more accurately treating the details of the electrostatics relevant to hyaluronan interacting with monoatomic cations in solution and polyatomic cations like lysine and arginine sidechains in protein binding partners. Critically, given the absence of experimental atomic-resolution structural biology methods directly capable of studying such systems, investigators will need to rely on indirect and/or low resolution experimental methods to corroborate observables from the simulation results as a means of validation. Indeed, a similar approach combining such experimental methods with simulations has been evolving to study intrinsically disordered proteins [208,209,210,211].

## Figures and Tables

**Table 1:** Complete list of PDB entries containing hyaluronan ^1^.

Description of Available Coordinates ^2^	Method	PDB ID [Reference]
8-mer from sodium fiber	Fiber diffraction	3HYA [49]
3-mer	Solution NMR	1HUA [37]
Two 2-mers + *Streptococcus pneumoniae* hyaluronate lyase	X-ray diffraction	1C82 [50]
2-mer + chondroitin AC lyase	X-ray diffraction	1HM3 [51]
6-mer + *Streptococcus pneumoniae* hyaluronate lyase	X-ray diffraction	1LOH [52]
4-mer + *Streptococcus pneumoniae* hyaluronate lyase	X-ray diffraction	1LXK [52]
6-mer + *Streptococcus agalactiae* hyaluronate lyase	X-ray diffraction	1LXM [53]
6-mer + *Streptococcus pneumoniae* hyaluronate lyase W291A/W292A mutant	X-ray diffraction	1N7Q [54]
6-mer + *Streptococcus pneumoniae* hyaluronate lyase W291A/W292A/F343V mutant	X-ray diffraction	1N7R [54]
8-mer	Solution NMR	2BVK [55]
7-mer + murine CD44 hyaluronan binding domain	X-ray diffraction	2JCQ [56]
7-mer + murine CD44 hyaluronan binding domain	X-ray diffraction	2JCR [56]
4-mer + murine CD44 hyaluronan binding domain	X-ray diffraction	4MRD [57]
2-mer + *Streptobacillus moniliformis* solute-binding protein	X-ray diffraction	6INZ [58]
Six 4-mers + bacteriophage hyaluronan lyase	X-ray diffraction	6WWX [59]
Two 4-mers + one 5-mer + two 6-mers + bacteriophage hyaluronan lyase	X-ray diffraction	6WXA [60]
Three 4-mers + three 8-mers + bacteriophage hyaluronan lyase	X-ray diffraction	6X3M [61]

^1^https://www.rcsb.org/ (accessed on 11 July 2022) “Advanced Search” for [“Oligosaccharide Component List BDP” AND “Oligosaccharide Component List NAG”] (n.b.: “BDP” = GlcA and “NAG” = GlcNAc, see https://www.wwpdb.org/data/ccd, accessed on 11 July 2022) followed by manual inspection of the resulting 20 hits for all structures actually containing hyaluronan yielded 14 entries. Two additional entries, 1C82 and 6INZ, both containing disaccharides, were found by substituting “GCD” (a deoxy/unsaturated analog of GlcA) for “BDP” in the search. 3HYA did not appear in “Advanced Search” results as that entry’s file lists “NGA” instead of “NAG” despite the fact that Model 2 of 2 in that entry contains GlcNAc (“NAG”) and not *N*-acetyl-β-D-galactosamine (GalNAc, “NGA”). 3HYA was found by a separate “Basic Search” for “hyaluronan”. ^2^ Describes coordinates as available in the PDB entry file for the “Biological Assembly”. The experimental systems may have contained longer hyaluronan polymers, but whose full coordinates are not available. Some terminal sugars may be analogs, e.g., deoxy/unsaturated. An “*x*-mer” contains *x* monosaccharide units, so, for example, a structure with *n* = 4 in Figure 1 is an “8-mer”. A limitation of this nomenclature is that, while allowing for polymers with an odd number of monosaccharide units, it does not uniquely identify which monosaccharide begins the sequence.

**Table 2:** Hyaluronan (*ϕ*_14_, *ψ*_14_) and (*ϕ*_13_, *ψ*_13_) dihedral angle values from PDB entries ^1^.

	*Index*	*i*		*i* + 1		*i* + 2		*i* + 3		*i* + 4		*i* + 5		*i* + 6		*i* + 7	
PDB ID	Chain	*ϕ*_14_	*ψ*_14_	*ϕ*_13_	*ψ*_13_	*ϕ*_14_	*ψ*_14_	*ϕ*_13_	*ψ*_13_	*ϕ*_14_	*ψ*_14_	*ϕ*_13_	*ψ*_13_	*ϕ*_14_	*ψ*_14_	*ϕ*_13_	*ψ*_13_
3HYA	A	−76.8	129.1	−52.2	−126.4	−80.8	127.6	−50.6	−118.6	−76.8	129.1	−52.2	−126.4	−80.8	127.6		
1HUA	model 2	−73.8	126.8	−79.8	−106.5												
1C82	B			−63.0	−116.5												
	C			−94.8	−127.6												
1HM3	D			−72.9	−126.1												
1LOH	B			−81.2	−141.2	−58.4	101.8	−60.4	−131.6	−67.7	51.2	−63.4	−158.5				
1LXK	B			−83.1	−139.3	−60.1	104.4	−58.5	−118.9								
1LXM	B			−98.7	−123.2	−57.5	102.3	−51.6	−117.5	−54.3	69.7	−61.4	119.2				
1N7Q	B			−94.4	−122.9	−63.2	80.0	−60.0	−115.6	−63.9	51.3	−67.0	−151.5				
1N7R	B			−89.2	−128.0	−63.2	97.5	−62.5	−132.1	−61.3	54.5	−68.1	−157.7				
2BVK	A	−71.2	126.0	−68.1	−110.3	−71.1	126.6	−68.1	−110.3	−71.1	126.6	−68.0	−110.3	−71.1	126.6		
2JCQ	B			−86.2	−154.1	−73.7	108.2	−69.0	−114.6	−88.6	133.7	−78.1	−119.4	−68.9	114.5		
2JCR	B			−91.9	−138.1	−71.2	107.9	−71.3	−117.1	−79.0	129.0	−82.5	−123.6	−75.4	103.6		
4MRD	B			−93.1	−151.0	−79.4	111.9	−67.5	−132.8								
6INZ	B			−92.5	−143.8												
6WWX	D			−79.2	−119.9	−96.2	123.5	−79.8	−131.3								
	E			−73.4	−106.0	−84.8	102.4	−95.7	−111.7								
	F			−77.9	−105.5	−91.4	105.7	−85.1	−110.2								
	G			−85.2	−116.1	−88.7	123.5	−86.2	−126.4								
	H			−79.7	−124.1	−93.6	128.0	−85.7	−131.7								
	I			−76.5	−108.2	−84.6	105.5	−73.2	−112.4								
6WXA	F			−83.8	−113.2	−91.7	111.0	−91.2	−97.0								
	H			−77.6	−106.5	−89.2	103.9	−66.3	−116.6								
	E			−71.9	−130.6	−90.1	128.0	−90.3	−124.2	−84.1	135.4						
	D			−83.1	−119.3	−93.2	120.1	−80.2	−130.8	−92.2	137.8	−66.7	−125.7				
	G			−82.5	−123.9	−88.1	125.8	−80.1	−123.4	−84.0	124.2	−64.5	−125.4				
6X3M	J			−78.6	−123.1	−88.5	109.7	−81.1	−130.5								
	N			−27.1	−125.3	−75.5	64.3	−116.6	−154.8								
	O			−64.0	−137.7	−102.8	74.1	−10.8	−112.1								
	K			−43.8	−124.8	−67.6	101.0	80.7	−130.0	−97.1	−45.6	−96.6	−107.3	88.7	166.0	−64.7	−146.5
	L			−63.2	−132.7	−64.5	−41.4	−132.4	−144.8	−110.4	−41.6	−72.3	−141.1	−67.8	109.7	−73.5	−137.0
	M			−146.9	155.6	17.9	147.3	−87.4	−128.9	−65.6	116.2	−64.1	−93.3	−115.1	97.6	−73.0	−135.6

^1^ Dihedral angle and index definitions are per Figure 1. Dihedral angle values are in degrees and were computed from PDB coordinates using Visual Molecular Dynamics (VMD) [62].

## Data Availability

Not applicable.
